# Prebiotic Factors Influencing the Activity of a Ligase Ribozyme

**DOI:** 10.3390/life7020017

**Published:** 2017-04-06

**Authors:** Fabrizio Anella, Christophe Danelon

**Affiliations:** Department of Bionanoscience, Kavli Institute of Nanoscience, Delft University of Technology, van der Maasweg 9, 2629 HZ Delft, The Netherlands; f.m.anella@tudelft.nl

**Keywords:** catalytic RNA, fatty acid, lipid vesicle, protocell, origins of life

## Abstract

An RNA-lipid origin of life scenario provides a plausible route for compartmentalized replication of an informational polymer and subsequent division of the container. However, a full narrative to form such RNA protocells implies that catalytic RNA molecules, called ribozymes, can operate in the presence of self-assembled vesicles composed of prebiotically relevant constituents, such as fatty acids. Hereby, we subjected a newly engineered truncated variant of the L1 ligase ribozyme, named tL1, to various environmental conditions that may have prevailed on the early Earth with the objective to find a set of control parameters enabling both tL1-catalyzed ligation and formation of stable myristoleic acid (MA) vesicles. The separate and concurrent effects of temperature, concentrations of Mg^2+^, MA, polyethylene glycol and various solutes were investigated. The most favorable condition tested consists of 100 mM NaCl, 1 mM Mg^2+^, 5 mM MA, and 4 °C temperature, whereas the addition of Mg^2+^-chelating solutes, such as citrate, tRNAs, aspartic acid, and nucleoside triphosphates severely inhibits the reaction. These results further solidify the RNA-lipid world hypothesis and stress the importance of using a systems chemistry approach whereby a wide range of prebiotic factors interfacing with ribozymes are considered.

## 1. Introduction

It has been postulated that catalytic RNA molecules, called ribozymes, have served essential functions at an earlier stage [1,2], before the transition to modern biology whereby DNA is the main informational polymer and protein enzymes are the catalysts. However, even in this RNA-centric hypothesis of the origins of life, it is clear that a myriad of other compounds and factors have influenced the folding, reactivity, selection, and evolution of ribozymes [3]. The molecular diversity of the prebiotic environment is exemplified by the wealth of sugar products generated in the formose reaction [4] or by the broad spectrum of compounds potentially available in a cyanosulfidic geochemical scenario [5]. There exists, however, a gap between the oversimplified experimental conditions used in the laboratory and the more complex repertoire of compounds presumably available on the early Earth [6].

Important factors that influence the activity of ribozymes include the temperature [7,8,9,10], Mg^2+^ concentration [11,12,13], fatty acid concentration [14], crowding agents [15,16,17], and carboxylate- or phosphate-containing solutes [18,19]. Of particular relevance is the role of divalent metal ions like Mg^2+^, as they assist three-dimensional folding of RNA [20,21] and can participate in the catalysis reaction [22,23]. On the other hand, Mg^2+^ mediates spontaneous cleavage of the RNA strand and disrupts fatty acid vesicles, whose role as primitive cell compartments has been advanced [24,25]. How ancestral RNA protocells [26,27] could emanate from these different factors mutually interacting remains a major challenge in prebiotic systems chemistry.

Here, we studied the activity of a truncated L1 ligase ribozyme, an RNA catalyst that forms a phosphodiester bond between the 3′-terminal nucleotide of a substrate and the ribozyme body itself [28]. This class of ribozyme is important if one considers that long RNA molecules showing higher level functionalities, like templated polymerization, may have arisen from the ligation of shorter oligomers, a reaction that could have been catalyzed by RNA ligase [29,30]. The L1 RNA ligase is tolerant to low Mg^2+^ concentrations, a condition compatible with fatty acid vesicle self-assembly and improved RNA strand stability against spontaneous hydrolysis [14]. However, the loss of ligation efficiency accompanying a reduction of Mg^2+^ concentration prompted us to explore ribozyme activity under a broader range of prebiotically plausible environmental conditions with the aim that a set of external co-factors could alleviate reactivity. We show that there exists a complex interplay between temperature, fatty acid concentration, and polyethylene glycol (PEG)-induced crowding, with enhancing or inhibiting effects depending on the precise conditions. Remarkably, low temperature counteracts the drop of activity at low Mg^2+^ concentrations, while intermediate concentrations of myristoleic acid (MA) provide the best compromise with respect to activity and ability to form vesicles. Moreover, carboxylate- or phosphate-containing solutes (amino acids, tRNA) decrease the efficiency of ligation, presumably by lowering the amount of free Mg^2+^. Although our experiments do not capture the full molecular complexity and environmental conditions on the early Earth, our findings on a new variant of the L1 RNA ligase emphasize that recapitulating in the laboratory the path from prebiotic chemistry to a ribozyme-based protocell asks for a global approach, in which RNA catalytic properties are investigated with a background of relevant compounds under various temperatures.

## 2. Experimental Section

### 2.1. Materials

All oligonucleotides were synthesized by Ella Biotech (Martinsried, Germany). The tRNA mixture derived from *Escherichia coli* was obtained from Roche. Myristoleic acid (MA, C14:1, ≥99% capillary GC) and the other solutes were purchased from Sigma-Aldrich Chemie B.V. (Zwijndrecht, The Netherlands).

### 2.2. Preparation of Ribozyme

The DNA template encoding for a truncated variant (56 ribonucleotides) of the short R8-9 of the L1 ligase ribozyme [28] was obtained by polymerase chain reaction (PCR). Briefly, 0.2 µM of template (5′-TTCTAATACGACTCACTATA*GGACCTCGGCGAAAGCTATTCGAAACGCGAAAGCACTTAG ATGTGAGGTTAGGTGC*-3′; the underlined sequence is the T7 promoter sequence, the ribozyme sequence is denoted in italics) were amplified using 0.2 µM of forward primer (5′-TCCTAATACGACTCACTATA-3′) and reverse primer (5′-GCACCTAACCTCACATCTAAG-3′) in a 50-µL reaction solution containing 200 µM of each dNTPs, 1×Phusion HF buffer, and 1 U Phusion DNA Polymerase (Thermo Scientific). The PCR product was analyzed using 2% agarose gel electrophoresis (100 V, 40 min), purified using the PCR Clean-up kit from Promega, and the amount and purity of amplified DNA were assessed by absorbance using a Nanodrop 2000c (Thermo Fisher Scientific, Waltham, MA, USA).

The L1 ligase RNA was transcribed from the purified template DNA using the RiboMAX Large Scale RNA Production System (Promega Benelux B.V., Leiden, The Netherlands). In vitro transcription was performed at 37 °C overnight in a 100-µL solution containing 500 ng of template DNA, 7.5 mM NTPs, and 10 µL of the T7 RNA polymerase mixture. The reaction was stopped on ice and the DNA template was degraded by DNaseI for 15 min at 37 °C. The produced RNA was purified using the RNeasy MinElute Clean up kit (QIAGEN), and its concentration and purity were determined by absorbance (NanoDrop 2000c). The integrity of the RNA was validated by 7 M urea denaturing 12% polyacrylamide gel electrophoresis (PAGE) (120 V, 100 min) after ethidium bromide staining for 15 min.

### 2.3. Ribozyme Activity Assay

The purified ribozyme (1 µM) was denatured by heating at 75 °C for 5 min and then slowly cooled down to room temperature for refolding. The ligation buffer (30 mM Tris-HCl pH 7.7, 100 mM NaCl, and the appropriate amount of MgCl_2_) was added and the reaction was triggered by the addition of 0.25 µM of the oligonucleotide substrate (5′-Cy5-AAAAAAAAAAAAAAAAAAAAAAUGCACU-3′) consisting of a 6-ribonucleotide sequence (underlined) and a poly(dA) tail fluorescently labelled at the 5′ end with the Cy5 dye. The reaction was incubated at various temperatures (namely, −20 °C, 4 °C, room temperature (21 °C), and 37 °C) and incubation times. The reaction was stopped by the addition of one volume equivalent of loading buffer (0.01% bromophenol blue, 13% ficoll (*w*/*v*), 7 M urea, 2 mM EDTA, 90 mM Tris-borate; ssRNA Ladder Loading Dye, New England Biolabs). The samples were denatured for 5 min at 75 °C and the reaction components were separated by denaturing PAGE (120 V, 100 min), and resolved by fluorescence imaging using a Typhoon Trio (GE Healthcare, Pittsburgh, PA, USA).

### 2.4. Ribozyme Activity Assay in the Presence of Citric Acid

Four different amounts of citric acid (Sigma-Aldrich) were pre-incubated with MgCl_2_ in a molar ratio of 4:1 and equilibrated for at least 1 h before use to avoid exposure of the ribozyme to unchelated Mg^2+^ ions. Then the purified ribozyme (1 µM final concentration) was treated as described in Section 2.3 and added to the reaction buffer (30 mM Tris-HCl, 100 mM NaCl, 1 mM MgCl_2_, pH 7.7) containing the complex citric acid/magnesium at the appropriate concentrations. The reaction was incubated at room temperature for 6 h and ligation efficiency was analyzed as described in Section 2.3.

### 2.5. Preparation of Micelles and Vesicles for Ribozyme Activity Assays

Fatty acid micelles were prepared by dispersing MA amphiphiles to a final concentration of 80 mM in milliQ water containing 90 mM of NaOH (pH 13). The solution was then vortexed briefly and tumbled overnight at room temperature. Argon was continuously flushed in the solution for 30 min before tumbling in order to ensure complete dissolution of any remaining oil droplets. In the experiments involving micelles, the solution was used immediately, after dilution to the appropriate concentrations in the reaction buffer. Vesicles were formed by adding the micelle stock solution to different volumes of the ligation buffer and tumbled overnight at room temperature. Before use, the particle size distribution was analyzed by dynamic light scattering (DLS). A 70-µL sample was added into a disposable microcuvette (ZEN0040, Malvern) and the measurements were carried out on a Zetasizer Nano ZS (Malvern, UK) operating at a scattering angle of 173° at room temperature. For each condition, the hydrodynamic radius profiles of three runs were analyzed. A representative curve is shown.

### 2.6. Ribozyme Activity Assay in the Presence of Fatty Acids

The purified L1 ribozyme treated as described in Section 2.3 was added at 1 µM final concentration to a solution containing appropriate amounts of MA in the ligation buffer. The solution was equilibrated for 30 min and the ligation reaction was triggered by adding 0.25 µM final concentration of Cy5-conjugated substrate. Ligation stop, sample denaturation, and visualization of the reaction product were performed as described in Section 2.3.

### 2.7. Ribozyme Activity Assay in the Presence of PEG

A 20% solution (*w*/*v*) of polyethylene glycol (PEG) was prepared in water. The purified ribozyme (1 µM final concentration) was added to the ligation buffer containing appropriate amounts of MgCl_2_ and PEG. The reaction was incubated at different temperatures for 6 h and the formation of the ligation product was analyzed as described in Section 2.3.

### 2.8. Ribozyme Activity Assay in the Presence of Various Solutes

Stock solutions of serine, histidine, aspartic acid, valine, tRNAs, and NTPs were prepared in water. The purified ribozyme (1 µM final concentration) was added to the ligation buffer supplemented with the appropriate amounts of solutes. The reaction was incubated at 4 °C for 6 h (unless otherwise indicated) and was analyzed as previously described.

### 2.9. Ribozyme Activity Assay in the Presence of a Mutated Substrate

A single-base mutated substrate (5′-Cy5-AAAAAAAAAAAAAAAAAAAAAA*UGCCCU*-3′, the 6-ribonucleotide stretch is denoted by italics, the position of the mutation with a “C” instead of an “A” is underlined) was used in some experiments, as indicated.

### 2.10. Gel Analysis

PAGE analysis was performed by quantifying the band intensities using the ImageJ software [31]. For every gel, we analyzed both the band intensities of the product (ligated substrate) and the unreacted substrate. For further quantitative analysis, as well as for the purpose of displaying representative gel data in the corresponding figures, we opted for the band (product versus unreacted ligand) that showed the most pronounced change of intensity across the conditions tested in that particular experiment. For instance, in the kinetics experiments with 1 mM Mg^2+^, the band intensity values corresponding to the reacted substrate significantly change across the different time points in a consistent manner over the three independent experiments, whereas the bands for the unreacted ligand show less noticeable (or no) differences. Instead, the bands of the unligated substrate were analyzed in the kinetics experiments with 10 mM Mg^2+^ for similar reasons. The fraction of unreacted substrate was then calculated either by directly measuring the band intensity of the unreacted substrate as (S/S_0_) or by measuring the band intensity of the product as (1 − P/S_0_), where P/S_0_ denotes the ratio of the amount of ligated substrate to that of the total substrate. The apparent ligation rate constant was determined by linear regression of the natural logarithm of S/S_0_ or (1 − P/S_0_) versus time. Error bars represent standard deviations.

### 2.11. Fluorescence Microscopy Imaging of Vesicles

A thin film of MA was prepared by evaporating the chloroform/methanol (80:20) solution overnight, followed by at least 1 h exposure to low pressure in a ConcentratorPlus (Eppendorf) at 30 °C. Vesicles were formed by rehydration of the film with 50 mM Tris-HCl pH 8.3, 1 mM MgCl_2_, 1 µM purified ribozyme, and 1 µM substrate to obtain a 10-mM final concentration of lipids. Samples were vortexed for about 1 min and then tumbled overnight a room temperature. Prior to imaging the vesicles were labeled using 1 µM rhodamine 6G (Sigma-Aldrich) as a lipid dye. A 10-µL droplet was squeezed into a homemade silicon chamber mounted onto a #1.5 glass coverslip. The samples were imaged using a fluorescence confocal microscope (A1+ from Nikon) equipped with a ×100 oil immersion objective, the 561-nm and 633-nm laser lines with appropriate dichroic mirrors and emission filters.

## 3. Results and Discussion

### 3.1. Low Temperature Decreases Mg^2+^ Requirement of a New Truncated L1 Ribozyme

**Figure 1:**
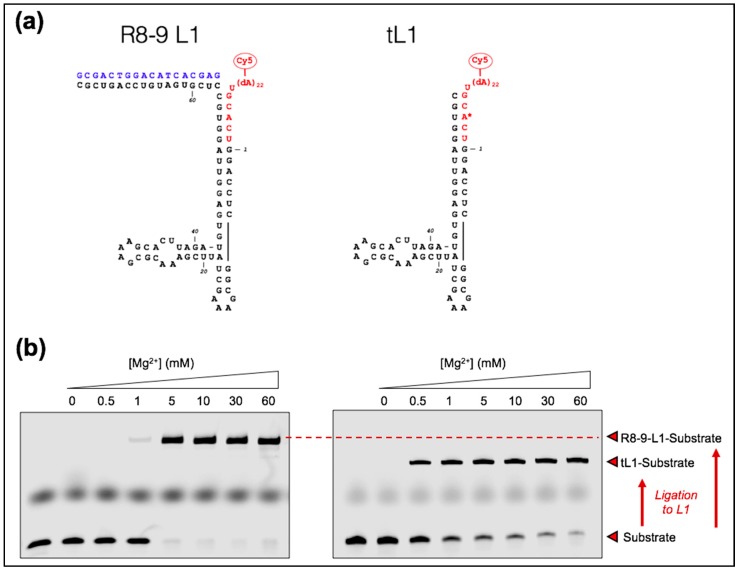
(**a**) Schematic of the R8-9 L1 and tL1 ribozymes. The image depicts the predicted secondary structures with the ribozyme body colored in black, the substrate in red, and the effector oligonucleotide in blue. The asterisk indicates that the A nucleobase of the canonical substrate is replaced by an C in the mutated substrate. (**b**) Polyacrylamide gel electrophoresis (PAGE) analysis of the ligation reaction mediated by the R8-9 L1 (left) and tL1 (right) ribozymes. Solutions containing 1 µM ribozyme, 0.25 µM substrate, and, in the case of R8-9 L1, 2 µM effector were incubated at the indicated Mg^2+^ concentration at room temperature for 3 h. The first lane of each gel is only loaded with the substrate as a reference.

The ribozyme used in this study is a truncated version (56 ribonucleotides) of the short R8-9 L1 ribozyme (74 ribonucleotides) [28]. The annealing region of the DNA effector was removed to simplify the reaction scheme, thus reducing the number of ligation partners from three to two, namely, the truncated ribozyme body and its oligonucleotide substrate. We called this truncated variant of the L1 ligase tL1 (Figure 1a). Activity of the ribozyme was assessed on polyacrylamide gel by measuring the ligation of a fluorescent substrate to the ribozyme body (Figure 1b). The tL1 ribozyme exhibits similar activity compared to the R8-9 ligase, suggesting that the effector-binding domain is not essential to ribozyme reactivity (Figure 1b). A larger fraction of converted substrate at 1 mM Mg^2+^ can even be seen.

**Figure 2:**
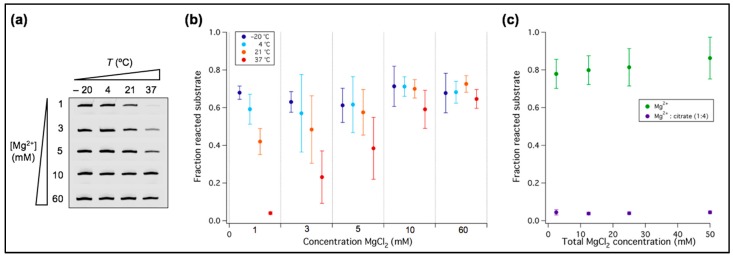
Mg^2+^ dependency of the tL1 activity as a function of temperature. (**a**) PAGE analysis of the ligation reaction. Only the bands corresponding to the ligated substrate are shown. One micromolar of tL1 and 0.25 µM of substrate were incubated at the indicated Mg^2+^ concentration and temperature for 6 h. (**b**) Quantitation of ligation efficiency expressed as the fraction of reacted substrate. Three independent experiments were performed for each condition; error bars represent standard deviations. (**c**) Quantitative analysis of the ligation reaction in presence of citric acid. Four different concentrations of Mg^2+^ were preincubated with citric acid in a 1:4 ratio before exposure to tL1 and substrate. Reactions were run at room temperature for 6 h and the fraction of ligated substrate was calculated. Three independent experiments were performed for each condition; error bars represent standard deviations.

Titrating the Mg^2+^ concentration we found that below 10 mM tL1 activity significantly drops (Figure 1b). We then asked whether temperature could modify the sensitivity of tL1 to Mg^2+^ concentration. This is an important aspect regarding the search of unifying conditions for both high ribozyme activity and fatty acid vesicle integrity. Ligation efficiency of tL1 and substrate was investigated at different Mg^2+^ concentrations and temperatures after 6 h reaction (Figure 2). When lowering the Mg^2+^ concentration from 60 mM to 1 mM, the amount of ligation product gradually decreases at 37 °C and 21 °C, whereas it remains nearly constant at 4 °C and −20 °C. At 60 mM Mg^2+^ the tL1 activity is independent of temperature. This result suggests that low-temperature together with 1 mM Mg^2+^ could provide a favorable environment for combining active tL1 ribozyme with fatty acid protocell models.

An alternative approach to minimize detrimental effects of Mg^2+^ with respect to fatty acid vesicle stability and RNA integrity against strand cleavage is to partially chelate Mg^2+^ with citric acid [32]. Coordinating Mg^2+^ to citrate enabled nonenzymatic templated RNA polymerization inside fatty acid vesicles [32]. Hence, we examined the role of citrate as a potential candidate to improve compatibility between ribozyme activity and vesicle experiments. When exposed to a 1:4 equivalent of Mg^2+^ and citrate, ligation by tL1 is completely inhibited for absolute concentrations of Mg^2+^ ranging from 2.5 mM to 50 mM (Figure 2c). This result indicates that more than three coordination sites of Mg^2+^ ions are required to fold tL1 in an active conformation or to catalyze ligation.

**Figure 3:**
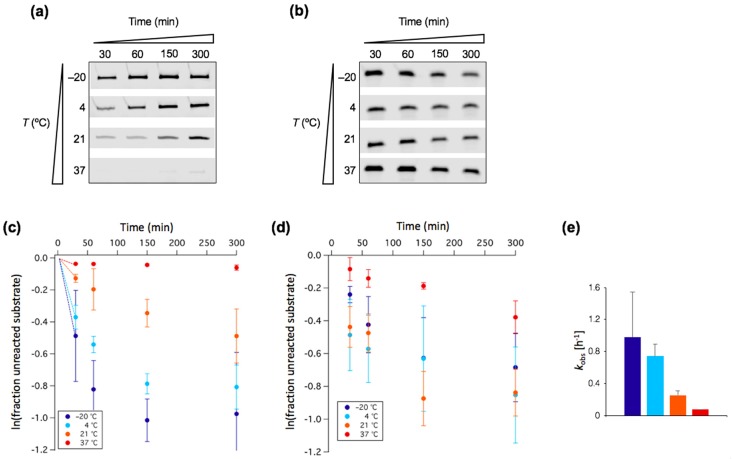
Kinetics of tL1-catalyzed ligation. (**a**,**b**) PAGE analysis of ligation reactions performed with [tL1] = 1 µM and [substrate] = 0.25 µM in the presence of 1 mM Mg^2+^ (**a**) or 10 mM Mg^2+^ (**b**). In (**a**) the product bands are shown, whereas the bands corresponding to the unreacted substrate are displayed in (**b**). The natural logarithm of the fraction of reacted substrate was calculated for the experiments under 1-mM Mg^2+^ (**c**) and 10-mM Mg^2+^ (**d**) conditions. Three independent experiments were performed for each condition; error bars represent standard deviations. The appended dashed lines in (**c**) are the initial slopes that were used to calculate the observed ligation rate constant of the fast kinetics component, *k*_obs_. (**e**) Values of *k*_obs_ under 1-mM Mg^2+^ condition. Color coding for the different temperatures is similar as in panel (**c**).

To gain quantitative insights on the kinetics of tL1-catalyzed ligation, we performed a time series analysis of the reaction at 1 mM and 10 mM Mg^2+^ concentrations (Figure 3a,b). We found that ligation deviates from monophasic kinetics (Figure 3c,d). The fast observed (or apparent) ligation rate constant, *k*_obs_, was estimated at 1 mM Mg^2+^ using the initial slope on the graph (Figure 3c). Values gradually drop from 0.98 ± 0.57 h^−1^ at −20 °C to 0.074 ± 0.002 h^−1^ at 37 °C (Figure 3e).

Throughout this study, the ribozyme was prepared under the same conditions (transcription, purification, refolding) to ensure that its initial conformation was identical at the start of all experiments. However, we do not exclude the possibility that within the pool of input ribozymes a fraction is not correctly folded [33] or folded in different conformations with varying degrees of reactivity regarding substrate binding and/or ligation. Such a conformational heterogeneity could explain the complex kinetics profile shown in Figure 3. Hence, the extracted *k*_obs_ values only report on the kinetics of the subset of tL1 with higher reactivity.

The observation that freezing temperature favors reactivity suggests that molecular diffusion is not the rate-limiting step of ligation. Instead, low temperature has a chaperone-like effect that stabilizes tL1 in an active conformation and can substitute Mg^2+^. It has been reported that the L1 ribozyme is flexible and can adopt many conformations [34]. Lowering temperature, just like increasing Mg^2+^ abundance, may potentiate ribozyme function by confining the structural exploration space around active conformers. Reduction of Mg^2+^ requirement as temperature decreases has previously been observed for the cis ligation of the hairpin ribozyme [35]. Substituting Mg^2+^ for low temperature without compromising reactivity offers the advantage of better compatibility with the formation of stable fatty acid vesicles and of reduced spontaneous RNA cleavage.

### 3.2. Characterization of Fatty Acid Micelles and Vesicles at 1 mM Mg^2+^

**Figure 4:**
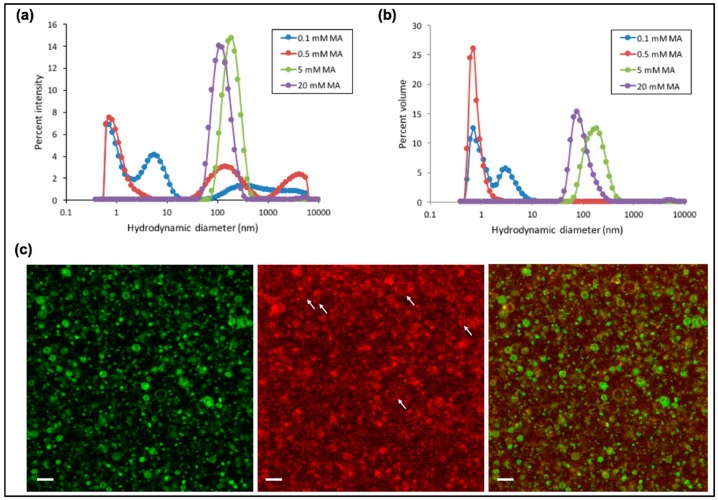
Dynamic light scattering (DLS) measurements performed at varying concentrations of myristoleic acid (MA) and reported as percent intensity (**a**) and percent volume (**b**) as a function of the hydrodynamic diameter. For each condition, a representative size distribution profile from at least two independent experiments (three runs each) is shown. (**c**) Fluorescence confocal microscopy images of fatty acid vesicles formed with 10 mM MA in Tris-HCl 50 mM, pH 8.3, 1 mM MgCl_2_, 1 µM substrate and 1 µM tL1. The white arrows point toward some liposomes with a diameter >0.5 µm which enables visualization of the substrate colocalizing with the vesicle membrane. Scale bars are 4 µm.

Motivated by our finding that tL1 retains full activity at ≤4 °C and ~50% at 21 °C in the presence of 1 mM Mg^2+^ compared to that with 60 mM Mg^2+^, we next examined the types of self-assembly structures formed at different concentrations of the MA fatty acid under such ligation conditions. Micelles preferentially form at 0.1 mM and 0.5 mM MA concentrations, while vesicles with an average diameter around 100–200 nm become predominant at 5 mM and 20 mM, as observed by DLS (Figure 4a,b). Additionally, fluorescence confocal microcopy reveals that a subset of vesicles exhibits a diameter >1 µm with a very distinct lumen (Figure 4c). Interestingly, fluorescence colocalization of the membrane dye and the Cy5-conjugated substrate suggests binding between the fatty acid bilayer and the oligonucleotide substrate free or ligated to the tL1 body. The exact nature of this interaction is unclear, but it presumably involves Mg^2+^ to bridge the negatively charged RNA and the fatty acid surface. Such interaction, if not inhibitory for tL1 activity, could form the basis for mutually reinforcing mechanisms between ribozymes and fatty acid bilayers.

### 3.3. Ribozyme Activity Profile as a Function of Fatty Acid Concentration Exhibits a Maximum

**Figure 5:**
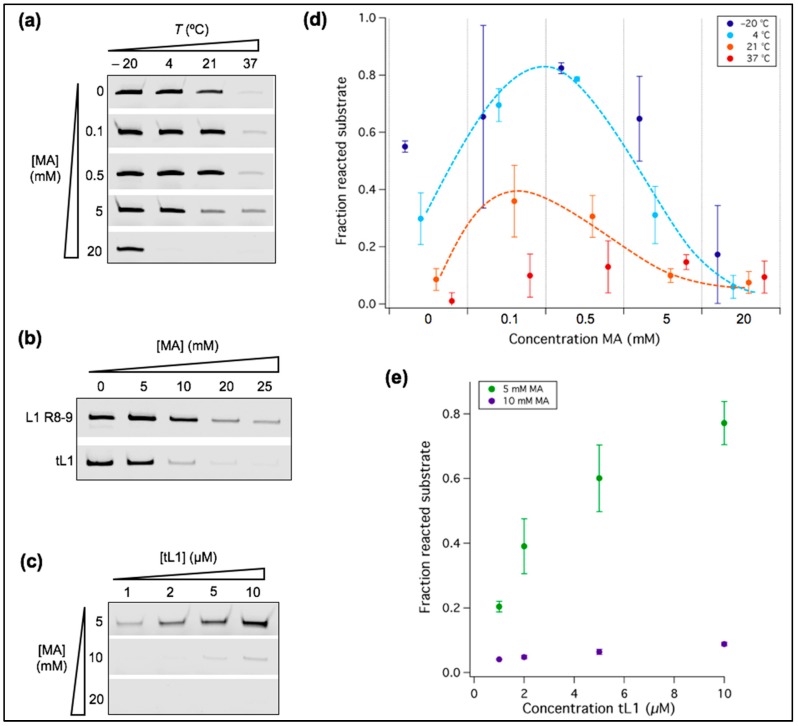
Temperature and MA concentration as factors regulating ribozyme activity. (**a**–**c**) PAGE analysis of the ligation reactions. Only the product band is displayed for each condition tested. Unless indicated otherwise, [Mg^2+^] = 1 mM, [ribozyme] = 1 µM, [substrate] = 0.25 µM, and ligation occurred for 6 h. (**b**) The reaction with either L1 R8-9 or tL1 was incubated at room temperature (21 °C) for 40 h. (**c**) The reaction was performed at room temperature for 6 h. (**d**) Quantitative analysis of the ligation reactions shown in (**a**). The fraction of unreacted substrate was measured. The dashed lines have been appended to guide the eye. Three independent experiments were performed for each condition; error bars represent standard deviations. (**e**) Quantitative analysis of the reactions shown in (**c**). The fraction of ligated substrate was measured and plotted as a function of the tL1 concentration. Three independent experiments were performed for each condition; error bars represent standard deviations.

We previously reported that at 37 °C the ligation yield by the R8-9 L1 ribozyme increases upon the addition of 5 mM MA, but decreases at higher concentrations [14]. Here, we further analyzed the effects of MA concentrations and temperature on the activity of tL1 at 1 mM Mg^2+^, a concentration compatible with the formation of micellar and vesicular lipidic structures (Figure 4). We observed that MA could either enhance or impair ribozyme activity depending on the fatty acid concentration and reaction temperature (Figure 5). The addition of MA micelles at 0.1 mM or 0.5 mM fatty acid concentrations leads to a gain of function at all temperatures tested (Figure 5a,d). Interestingly, 5 mM MA gives rise to a higher ligation yield at 37 °C, as already observed with the original L1 R8-9 ribozyme [14], but not at lower temperatures (Figure 5a,d). Because such an enhancement is also observed in the presence of micelles ([MA] ≤ 0.5 mM), it cannot be attributed to bilayer membrane exposure. In contrast, MA concentrations of 5 mM and 20 mM (corresponding to stable vesicles) have an inhibitory effect at room temperature or lower (Figure 5a,d), and this effect is more pronounced on tL1 compared to the original L1 ligase (Figure 5b). We hypothesized that depletion of Mg^2+^ by fatty acids could explain this phenomenon. To test this hypothesis, increasing concentrations of tL1 were used, with 5 mM up to 20 mM MA at fixed 1 mM Mg^2+^, with the reasoning that a higher concentration of tL1 would only result in more ligation product if the Mg^2+^ concentration was not limiting. While 10 mM and 20 mM MA are strongly inhibitory at tL1 concentrations ranging from 1 to 10 µM, a clear increase in the amount of product is observed at 5 mM MA (Figure 5c,e), suggesting that enough Mg^2+^ remains available to support activity of up to 10 µM tL1. Another possible mechanism of lipid-mediated inactivation is direct interaction between the fatty acid structures and tL1 or its substrate (Figure 4c), which may cause RNA misfolding or hindered accessibility of the ligand binding site.

Together, these results reveal the complex interplay between the effects of temperature and fatty acid concentration, which leads to nonmonotonic activity profiles whose optimum is shifted to higher MA concentrations when temperature increases. Moreover, the results favor a low-temperature condition for coexistence of stable vesicles and substantial ribozyme activity.

### 3.4. PEG Acts as a Positive or Negative Regulator of Ribozyme Activity Depending on Temperature

**Figure 6:**
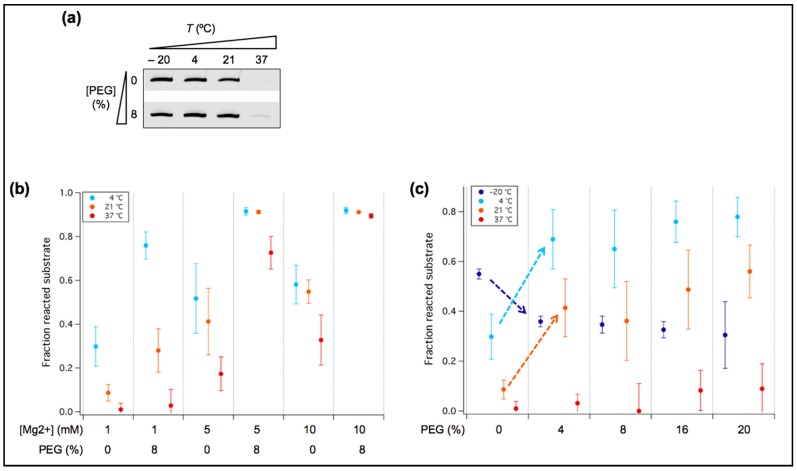
Influence of crowding agent on ribozyme activity. (**a**) PAGE analysis of the ligation reaction with or without 8% PEG. One micromolar of tL1 and 0.25 µM of substrate were incubated in the presence of 1 mM Mg^2+^ for 6 h at the indicated temperatures. Only the product band is displayed for each condition. (**b**) Quantitative analysis of the ligation reactions performed at various Mg^2+^ concentrations in the presence or absence of 8% PEG. One micromolar of tL1 and 0.25 µM of substrate were incubated in the presence of 1, 5 or 10 mM Mg^2+^ for 6 h at the indicated temperatures. The fraction of unreacted substrate was measured. Three independent experiments were performed for each condition; error bars represent standard deviations. (**c**) Quantitative analysis of the ligation reaction as a function of PEG concentration. One micromolar of the ribozyme and 0.25 µM of the substrate were incubated in presence of 1 mM Mg^2+^ and varying amounts of PEG for 6 h at the indicated temperatures. The fraction of unreacted substrate was measured. Three independent experiments were performed for each condition; error bars represent standard deviations. The dashed arrows guide the eye to the marked increase or decrease of tL1 activity upon PEG addition.

Besides temperature and fatty acids, another factor that may influence ribozyme activity is molecular crowding. In particular, we asked whether crowding could reduce the Mg^2+^ requirement, including at temperatures >4 °C. Titration with PEG, a prototypical crowding agent, indicates that maximum effects on tL1 activity is reached at 8% (*w*/*v*) PEG at 1 mM Mg^2+^ in the range of temperatures tested (Figure 6a,c). Whereas the presence of PEG is inhibitory at −20 °C, it has a positive effect at higher temperatures, with marked improvement at 4 °C and at room temperature. We therefore further investigated the role of PEG using 8% (*w*/*v*) working concentration and positive temperatures (Figure 6b). At 4 °C and 21 °C, PEG-induced yield enhancement is also pronounced at Mg^2+^ concentrations higher than 1 mM, with almost total substrate ligation observed at 10 mM Mg^2+^, denoting a cumulative effect of crowding and Mg^2+^ (Figure 6b). However, at 1 mM Mg^2+^ and 37 °C, no significant effect of PEG was measured (Figure 6b).

Increased ribozyme activity in crowded solutions has already been reported [15]. Molecular crowders like PEG may stabilize the folded structure of tL1 through excluded volume effects, populating its functional conformation. Another outcome of the presence of PEG is the increase of the effective concentration of Mg^2+^ in solution, which may also stabilize the ribozyme in its most compact, active state. The fact that no increase of ligation yield was observed upon PEG addition at 1 mM Mg^2+^ and 37 °C, two conditions that perturb ribozyme folding into compact structures, suggests that crowding effects are not strong enough to compensate the loss of functional folding. Additionally, the inhibitory effect of PEG observed at −20 °C with 1 mM Mg^2+^ (Figure 6c) is presumably due to the slow diffusion of tL1 and its substrate, making bimolecular association the rate limiting step of the reaction. Though PEG itself is likely not a prebiotic molecular crowder, its effects could resemble that generated by some crowding (micro)environments that were present on the early Earth, such as in mineral cavities [36,37].

### 3.5. Lowering the Affinity between Ribozyme and Substrate Leads to Severe Loss of Reactivity

**Figure 7:**
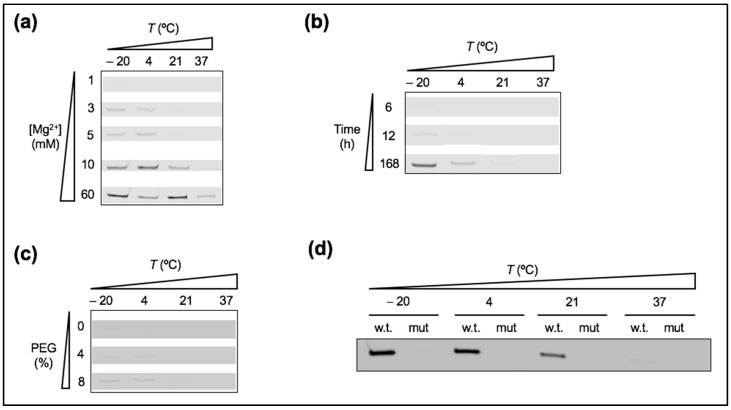
Ribozyme activity in presence of the mutated substrate. (**a**) PAGE analysis of the ligation reaction as a function of temperature and Mg^2+^ concentration. One micromolar of the ribozyme and 0.25 µM of the mutated substrate were incubated for 6 h. The indicated Mg^2+^ concentration was added. (**b**) PAGE analysis of the ligation kinetics performed with 1 µM tL1, 0.25 µM of the mutated substrate, and 1 mM Mg^2+^. The different temperatures and incubation times are indicated. (**c**) PAGE analysis of the ligation reaction performed with 1 µM tL1, 0.25 µM of the mutated substrate, 1 mM Mg^2+^, and incubated for 6 h at the indicated temperatures and PEG concentrations. (**d**) PAGE analysis of the ligation reaction performed with 1 µM of tL1, 0.25 µM of either the ‘wild-type’ or mutated substrate, 1 mM Mg^2+^, and 5 mM MA, and incubated for 6 h at the indicated temperatures. In all gels (**a**–**d**), only the bands of the product are shown.

We evaluated how much weakening the interaction between the tL1 ribozyme body and its substrate affects the yield of ligation, and to what extent a drop of activity could be restored by temperature, PEG, or MA vesicles. We used a mutated substrate with single-nucleotide mismatch (Figure 1a). The ability of tL1 to ligate the low-affinity substrate when exposed to various environmental conditions was analyzed at different Mg^2+^ concentrations. Under low Mg^2+^ conditions, the fraction of ligated substrate was very low (<0.1) across the full range of tested temperatures (Figure 7a), including when the reaction solution was supplemented with 8% PEG (Figure 7c), or when the reaction duration was prolonged up to 7 days (Figure 7b). The addition of 5 mM MA in the form of vesicles did not increase yield after 6 h reaction (Figure 7d). Only when Mg^2+^ concentration was raised above 3 mM could the ligation product be detected (Figure 7a) with a maximum fraction of reacted substrate of ~0.15 at 60 mM Mg^2+^ and −20 °C. These results show that the drop of product formation arising from low-affinity binding cannot be overcome by external factors that contribute to stabilize tL1 in an active conformation. Because substrate binding may precede ribozyme folding [38], the low-affinity ligand might fail to convert tL1 into an active conformation.

### 3.6. Ribozyme Activity Collapses in the Presence of Anionic Solutes

There exists a gap between the conventional oversimplified—practically convenient—laboratory conditions, where a particular mechanism is studied in an artificial medium containing solely the expected interacting partners, and the molecular diversity of the prebiotic environments. Mimicking the abiotic molecular complexity when investigating particular processes, here the ligation by the tL1 ribozyme, is indeed of direct relevance as background solutes may directly or indirectly interfere with the intended reactions.

**Figure 8:**
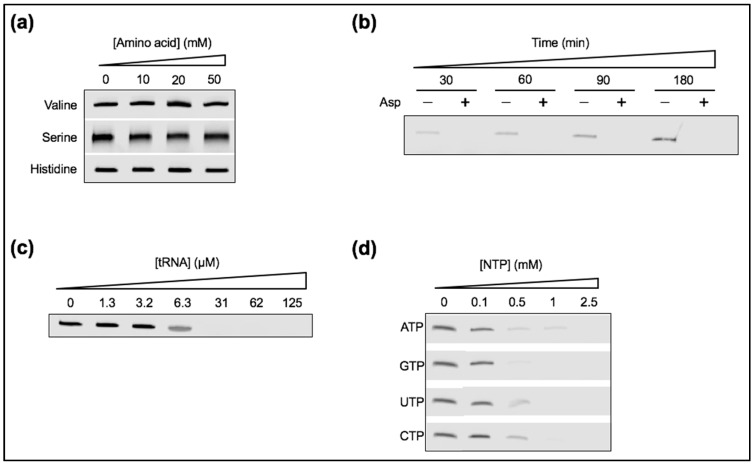
Ribozyme activity in the presence of various zwitterionic or negatively charged chemicals. Unless otherwise indicated, the reactions consisted of 1 µM ribozyme, 0.25 µM substrate, and 1 mM Mg^2+^, and were performed at 4 °C for 6 h. In these conditions, and in the absence of additional solutes, ~60% of substrate can be ligated (Figure 2b). The ligation reaction was supplemented (**a**) with valine, serine, or histidine; (**b**) with 10 mM aspartic acid (or not) at the indicated time points; (**c**) with a tRNA mixture at the indicated concentrations; and (**d**) with different NTPs at the indicated concentrations. The gel images show the product bands only.

To bridge this gap, we decided to supplement the tL1 reaction solution with a number of biochemically relevant solutes. Various amino acids or anionic chemicals, including a mixture of tRNAs and ribonucleotides, were added, while Mg^2+^ concentration was set to 1 mM and temperature to 4 °C. Though tRNAs are probably the product of biological evolution, we used them as an example of long RNA that can fold into tight secondary structures, as this might have been the case for structurally complex polymerase ribozymes. We found that tL1-mediated ligation was severely impaired in the presence of all negatively charged solutes (Figure 8b–d, see also the effect of citric acid in Figure 2c), presumably a consequence of their interaction with Mg^2+^. In contrast, the addition of zwitterionic valine, serine, or histidine was neutral to ribozyme activity (Figure 8a). These results emphasize the bias introduced by studying catalytic activity of RNA in an insulated manner (i.e., isolated from the expected diversity of prebiotic compounds). They also shed light on new opportunities provided by the presence of carboxy- or phosphate-containing molecules that compete for metal ions with the carboxylate group of fatty acids and, in turn, could deplete Mg^2+^ beyond its detrimental concentration for vesicle stability. It has been suggested that Mg^2+^ concentration in the primordial ocean was around 10 mM [39], an amount exceeding that compatible with the formation of vesicles from the most prebiotically plausible fatty acids. Hence, Mg^2+^-sequestrating chemicals like those used in the present study could have lowered the effective concentration of Mg^2+^ to a few millimolar only, which is compatible with both RNA ligation and fatty acid vesicle integrity.

## 4. Conclusions

In this study, we quantified the influence of various external factors on a new truncated variant of the L1 ligase ribozyme. While high Mg^2+^ concentration consistently leads to higher reactivity, particular attention was paid to the role of temperature and fatty acid concentrations that individually exhibit opposing effects depending on the specific conditions. We found that a temperature of 4 °C, 5 mM MA amphiphiles, and 1 mM Mg^2+^ best reconciles ribozyme activity with the formation of fatty acid vesicles, whereas the addition of Mg^2+^-chelating solutes inhibits ligation. Because evolution is inextricably linked to environmental conditions, the above considerations are important for the emergence of more effective and specialized ribozymes encapsulated inside vesicle compartments, where they can adapt to relevant evolutionary challenges. Finally, we postulate that these conclusions are not unique to the L1 ligase, but that a large spectrum of ribozymes could similarly be affected by such prebiotic factors.

## References

[1] Crick F.H.C. (1968). The origin of the genetic code. J. Mol. Biol..

[2] Gilbert W. (1986). The RNA world. Nature.

[3] Higgs P.G., Lehman N. (2015). The RNA world: Molecular cooperation at the origin of life. Nat. Rev. Genet..

[4] Butlerow A. (1861). Formation synthétique d’une substance sucrée. C. R. Acad. Sci..

[5] Patel B.H., Percivalle C., Ritson D.J., Duffy C.D., Sutherland J.D. (2015). Common origins of RNA, protein and lipid precursors in a cyanosulfidic protometabolism. Nat. Chem..

[6] Bad J.L. (2004). How life began on Earth: A status report. Earth Planet. Sci. Lett..

[7] Mutschler H., Lochner A., Holliger P. (2015). Freeze-thaw cycles as drives of complex ribozyme assembly. Nat. Chem..

[8] Frommer J., Appel B., Müller S. (2015). Ribozymes that can be regulated by external stimuli. Curr. Opin. Biotechnol..

[9] Vlassov A.V., Johnston B.H., Landweber L.F., Kazakow S.A. (2004). Ligation activity of fragmented ribozyme in frozen solution: Implication for the RNA world. Nucleic Acids Res..

[10] Vlassov A.V., Kazakov S.A., Johnston B.H., Landweber L.F. (2005). The RNA world on ice: A new scenario for the emergence of RNA information. J. Mol. Evol..

[11] Szostak J.W. (2012). The eightfold path to non-enzymatic RNA replication. J. Syst. Chem..

[12] Inoue A., Takagi Y., Taira K. (2004). Importance in catalysis of a magnesium ion with very low affinity for a hammerhead ribozyme. Nucleic Acids Rese..

[13] Amotov S., Nishikawa S., Taira K. (1996). Dependence on Mg^2+^ ions of the activities of dimeric hammerhead minizymes. FEBS Lett..

[14] Anella F., Danelon C. (2014). Reconciling ligase ribozyme activity with fatty acid vesicles stability. Life.

[15] Desai R., Kilburn D., Lee H.T., Woodson S.A. (2014). Increased Ribozyme activity in crowded solutions. J. Biol. Chem..

[16] Paddle B.P., Rueda D. (2014). Molecular crowding accelerates ribozyme docking and catalysis. J. Am. Chem. Soc..

[17] Strulson C.A., Yennawar N.H., Rambo R.P., Bevilacqua P.C. (2013). Molecular crowding favours reactivity of a human ribozyme under physiological ionic conditions. Biochemistry.

[18] Sengupta A., Sung H.L., Nesbitt D.J. (2016). Amino acid specific effects on RNA tertiary interactions: Single-molecule kinetic and thermodynamic studies. J. Phys. Chem. B.

[19] Nakano S., Kitigawa Y., Miyoshi D., Sugimoto N. (2015). Effects of background anionic compounds on the activity of the hammerhead ribozyme in Mg2+-unsaturated solutions. J. Biol. Inorg. Chem..

[20] Misra V.K., Draper D.E. (2002). The linkage between magnesium binding and RNA folding. J. Mol. Biol..

[21] Bowman J.C., Lenz T.K., Hud N.V., Williams L.D. (2012). Cations in charge: Magnesium ions in RNA folding and catalysis. Curr. Opin. Struct. Biol..

[22] Hamper A., Cowan J.A. (1997). A unique mechanism for RNA catalysis: The role of metal cofactor in hairpin ribozyme cleavage. Chem. Biol..

[23] Hanna R., Doudna J.A. (2000). Metal ions in ribozyme folding and catalysis. Curr. Opin. Chem. Biol..

[24] Chen I.A., Walde P. (2010). From self-assembled vesicles to protocells. Cold Spring Harbor Perspect. Biol..

[25] Attwater J., Hollinger P. (2014). A synthetic approach to abiogenesis. Nat. Methods.

[26] Hanczyc M.M., Fujikawa S.M., Szostak J.W. (2003). Experimental models of primitive cellular compartments: Encapsulation, growth and division. Science.

[27] Chen I.A., Salehi-Ashtiani K., Szostak J.W. (2005). RNA catalysis in model protocell vesicles. J. Am. Chem. Soc..

[28] Robertson M.P., Hesselberth J.R., Ellington A.D. (2001). Optimization and optimality of a short ribozyme ligase that joins non-Watson-Crick base pairings. RNA.

[29] Robertson M.P., Scott W.G. (2007). The structural basis of ribozyme-catalyzed RNA assembly. Science.

[30] Joyce G.F. (2007). A glimpse of biology's first enzyme. Science.

[31] Schneider C.A., Rasband W.S., Eliceiri K.W. (2012). NIH Image to ImageJ: 25 years of image analysis. Nat. Methods.

[32] Adamala K., Szostak J.W. (2013). Nonenzymatic template-directed RNA synthesis inside model protocells. Science.

[33] Schmitt T., Lehman N. (1999). Non-unity molecular heritability demonstrated by continuous evolution in vitro. Chem. Biol..

[34] Giambasu G.M., Lee T.S., Scott W.G., York D.M. (2012). Mapping L1 Ligase ribozyme conformational switch. J. Mol. Biol..

[35] Kazakov S.E., Balatskaya S.V., Johnston B.H. (2006). Ligation of the hairpin ribozyme in cis induced by freezing and dehydration. RNA.

[36] Hansma H.G. (2014). The power of crowding for the origins of life. Orig. Life Evol. Biosph..

[37] Mast C.B., Schink S., Gerland U., Braun D. (2013). Escalation of polymerization in a thermal gradient. Proc. Natl. Acad. Sci. USA.

[38] Glasner M.E., Bergman N.H., Bartel D.P. (2002). Metal ion requirements for structure and catalysis of an RNA ligase ribozyme. Biochemistry.

[39] Mulkidjanian A.Y., Bychkov A.Y., Dibrova D.V., Galperin M.Y., Koonin E.V. (2012). Origins of first cells at terrestrial, anoxic geothermal fields. Proc. Natl. Acad. Sci. USA.

